# Bunch microclimate influence amino acids and phenolic profiles of Pinot noir grape berries

**DOI:** 10.3389/fpls.2023.1162062

**Published:** 2023-06-07

**Authors:** Romy Moukarzel, Amber K. Parker, Olaf J. Schelezki, Scott M. Gregan, Brian Jordan

**Affiliations:** ^1^ Department of Wine, Food and Molecular Biosciences, Lincoln University, Lincoln, New Zealand; ^2^ Ministry of Primary Industries, Christchurch, New Zealand

**Keywords:** Pinot noir, grapevine, temperature, amino acids, phenolic compounds, anthocyanins

## Abstract

**Introduction:**

The increase of temperature due to climate change at different phenological stages of grapevine has already been demonstrated to affect accumulation of primary and secondary metabolites in grape berries. This has a significant implication for Pinot noir especially in New Zealand context as these compounds can have direct and indirect effects on wine quality.

**Methods:**

This study investigates how varying bunch microclimate through changes in temperature applied at veraison stage can affect: fresh weight, total soluble solids, the accumulation of anthocyanins, total phenolics and amino acids of the grape berries. This was studied over two growing seasons (2018/19 and 2019/20) with Pinot noir vines being grown at two different temperatures in controlled environment (CE) chambers. The vines were exposed to 800 µmol/m2/s irradiance with diurnal changes in day (22°C or 30°C) and night (15°C) temperatures. This experimental set up enabled us to determine the accumulation of these metabolite at harvest (both seasons) and throughout berry development (second season).

**Results and discussion:**

The results showed that berry weight was not influenced by temperature increase. The total soluble solids (TSS) were significantly increased at 30°C, however, this was not at the expense of berry weight (i.e., water loss). Anthocyanin content was reduced at higher temperature in the first season but there was no change in phenolic content in response to temperature treatments in either season. The concentrations of total amino acids at harvest increased in response to the higher temperature in the second season only. In addition, in the time course analysis of the second season, the accumulation of amino acids was increased at mid-ripening and ripening stage with the increased temperature. Significant qualitative changes in amino acid composition specifically the α-ketoglutarate family (i.e., glutamine, arginine, and proline) were found between the two temperatures.

**Significance:**

This study is the first to provide detailed analysis and quantification of individual amino acids and phenolics in Pinot noir in response to changes in temperature applied at veraison which could aid to develop adaptation strategies for viticulture in the future.

## 1 Introduction

Grapevine productivity and berry quality are highly dependent on abiotic factors (i.e., temperature) which can affect the suitability of varieties for the growing regions (Wu et al., 2019). It is predicted that the surface temperature will increase between 2 – 4.8°C by the year 2035 (IPCC, 2018). In recent decades, earlier harvest dates have been observed when temperatures have increased significantly (Schultz and Stoll, 2010; Cook and Wolkovich, 2016; van Leeuwen and Darriet, 2016; García de Cortázar-Atauri et al., 2017; van Leeuwen and Destrac, 2017; Labbé, 2019). It is anticipated that the effects of projected increasing temperatures and changes in precipitation patterns on grape growing regions would result in significant biochemical and physiological alterations to the vines, changing potential land use suitability or quality of wines (Meehl et al., 2007; Malheiro et al., 2010; Hannah et al., 2013; van Leeuwen et al., 2019; Santos et al., 2020). Even with the increasing knowledge on management practices to control and manipulate grapevine growth and development, understanding these climate change effects on grape production and berry quality are central to then developing adaptation strategies (Jones, 2006; Pellegrino et al., 2014; Pons et al., 2017; Drappier et al., 2019; van Leeuwen et al., 2019).

Abiotic factors are shown to control the synthesis and degradation of primary (sugars, amino acids etc.) and secondary (phenolic compounds) metabolites through the regulation of their biosynthetic pathways at different stages of berry development (Jordan, 2017; Rienth et al., 2021). Temperature is one of the abiotic factors that greatly influences the composition and concentration of primary and secondary metabolites in the berries and is the most decisive factor in determining wine quality and typicality (van Leeuwen et al., 2004; Deluc et al., 2007; Mira de Orduña, 2010; Sadras et al., 2013; Luchaire et al., 2017). For most grapevine varieties, the optimum temperature during grape maturation stage for the ideal formation of aroma compounds is between 20 and 22°C (Almaraz, 2015). According to other studies, temperature of 30°C seems to be the key point for changes in primary and secondary metabolism (Mori et al., 2005; Downey et al., 2006).

Sugar concentration is a key quality component in grapes that determines the alcohol concentration in the final wine. The increase of temperature due to climate change has led to an increase in sugar concentrations at harvest (Petrie and Sadras, 2008; Webb et al., 2012; Hall et al., 2016; van Leeuwen and Darriet, 2016; Parker et al., 2020). For example, an increase in total soluble solids (TSS) was observed when Cabernet Sauvignon berries were exposed to day temperature of 30°C over the whole period of berry development (Buttrose et al., 1971). Moreover, a strong correlation was observed between sugar accumulation and temperature increase in Cardinal and Pinot noir until 35°C (Kliewer and Torres, 1972). At temperatures exceeding 30°C berry shriveling, or sugar accumulation disorder may occur, altering parameters such as titratable acidity, pH, and anthocyanin (Greer and Weston, 2010; Venios et al., 2020; Deloire et al., 2021; Xyrafis et al., 2022). This has led to the desynchronization of sugar with other metabolites such as higher sugar and lower organic acid concentrations, as well as changed the phenolic and aroma composition (van Leeuwen and Destrac, 2017).

The rise in temperature disrupts several metabolic pathways causing an alteration in the biosynthesis of essential compounds that are crucial for the grape must quality (Blancquaert et al., 2019). Fruit components such as anthocyanins have been demonstrated to be particularly sensitive to temperature (Tarara et al., 2008; Poni et al., 2018). A recent study showed that elevated temperature (28°C/18°C, day/night) applied to grapevine cuttings from fruit set to maturity under controlled conditions hastened berry development, with a greater impact on anthocyanin, color, and titratable acidity before the onset of ripening. The concentration of anthocyanins, color intensity and titratable acidity was reduced compared to the lower temperature treatment (24°C/14°C day/night) (Arrizabalaga-Arriazu et al., 2020). Temperatures above 27°C applied at pre-veraison stage were proven to induce low color, and reduced ABA and anthocyanin biosynthetic gene levels irrespective of light conditions in Koho grapes (Shinomiya et al., 2015). A reduction in color formation is noticeable when the temperature exceeded 30°C, and when the temperature between the period of flowering and veraison is over 37°C, an increase in volatilization of aroma compounds and a decrease in color are observed (Neethling et al., 2017; Bernardo et al., 2018). High temperatures of 35 and 42°C applied one week before veraison have been shown to decrease the total amount of anthocyanins in berries, as well as change their composition (Pastore et al., 2017). Other studies found that high temperatures equal to 35°C immediately prior to veraison in Cabernet Sauvignon resulted in an inhibition of mRNA transcription of anthocyanin biosynthetic genes (Mori et al., 2007). High temperature above 35°C at veraison prevented anthocyanin accumulation in berry skins of ‘Aki Queen’ grapevines, but high temperature applied prior to veraison had no effect on the anthocyanin accumulation (Koshita et al., 2015). Recently a significant decrease of anthocyanins in Pinot noir pigment content under high temperatures (35°C) at veraison was associated with the decrease in malvidin (de Rosas et al., 2022). On the other hand, tannins and flavan-3-ols, were less affected by temperature but showed an increase in concentration in response to UV exposure (Spayd et al., 2002). Other studies on the aromas and their precursors of fruity and floral nuances (monoterpenes) highlight the positive effects of higher temperatures (25-30°C) during ripening but also their negative effect on fruit metabolism whenever they are excessively high above 35°C) (van Leeuwen and Darriet, 2016; Pons et al., 2017). In an *In vitro* study, high temperature (40°C) considerably reduced flavonols (Degu et al., 2016). Hence, these conflicting results suggest more research is required to investigate the specific anthocyanin and phenolic responses in Pinot noir.

In grapes, free amino acids are the major nitrogenous compounds and are the starting precursors for important secondary compounds in grapes including phenolic compound, methoxypyrazines, thiols, esters, higher alcohols, flavonols, and anthocyanins (Guan et al., 2017). Elevated temperatures (28°C-35°C) have been reported to increase total amino acids in grapevines (Lecourieux et al., 2017; Torres et al., 2017; Wu et al., 2019). However, other sources have reported that ambient temperature (28°C/18°C, day/night) reduced total amino acid concentration two weeks after mid-veraison and seemed to delay amino acids maturity (Arrizabalaga-Arriazu et al., 2020). The abundance of amino acids from the glutamate family was reduced with heated treatment in Cabernet Sauvignon (Wu et al., 2019). However, other studies showed an increase in the accumulation of glutamate family in Shiraz berries with elevated temperature (35/20°C, day/night) at veraison and mid-ripening under controlled environment (Sweetman et al., 2014).

The forementioned research has reported anthocyanin and amino acid profiles in response to increasing temperatures, however, very few studies have explored the impact of climate change on the importance of color and quality parameters of Pinot noir (Kliewer and Torres, 1972; Dimitrovska et al., 2011; Lee and Skinkis, 2013; de Rosas et al., 2022). Furthermore, limited work has been attributed to study the impact of specific temperatures on the accumulation of primary and secondary metabolites in Pinot noir grape berries. Experimentation in controlled environments (growth chambers) enables the light or temperature environment to be controlled and manipulated to study their effects. Therefore, the main goal of this work was to investigate how varying bunch microclimate through changes in temperature in controlled environments, affect grape berry physiology and the subsequent formation of anthocyanin, total phenolic and amino acids of Pinot noir grapes.

## 2 Materials and methods

### 2.1 Preparation of plant material

Two bunch microclimate-controlled environment experiments were conducted in 2018/19 and 2019/20 respectively at Lincoln University, New Zealand (43.6434° S, 172.4678° E) with the *V. vinifera* cv. Pinot noir. In 2018/19, fruit-bearing cuttings (*Vitis vinifera* L. cv. Pinot noir clone 10/5) were collected mid-winter from the Lincoln University vineyard. The canes were trimmed to eight nodes before being wrapped in wet paper and stored at 4°C in the dark in enclosed plastic bags until use. Six node cuttings were prepared using the method described by Mullins and Rajasekaran (1980). Root growth and initial shoot growth was established using a heating block (25°C) on which the trays of cuttings were placed. The shoot from the most apical node was preferentially selected, unless it was weak or did not bear an inflorescence, and in this case the next most apical shoot was selected. The remaining nodes along the shoot were disbudded, and the main retained shoot was tipped above the most basal inflorescence to encourage growth of a lateral shoot for leaf area as per Mullins and Rajasekaran (1980). In the case where two inflorescences had appeared, the apical inflorescence was removed. Cuttings that had successful root growth were transferred into 2 L pots late November into potting mix (horticultural bark, perlite, potting mixture with fertilizer: Osmocote 10-10-10, horticultural lime, Micromax trace elements and Hydraflo). As for the second experiment (2019/20), commercially obtained vines of Pinot noir Abel clone grafted onto 3309C (Coudrec) rootstock were used instead of locally produced first year Mullins vines. These were planted in 4 L pots in mid-November 2019 and after budburst and initial shoot growth, the strongest shoot was selected (in the case when both buds burst on the vine) and left to grow and develop. The basal inflorescence was retained per shoot.

### 2.2 Controlled environment experimental set up

In the first experiment (2018/19), the plants were moved from the greenhouse just before veraison (13 February 2019) into 2 x CE rooms (15 plants per room) with day/night temperatures of 22°C/15°C. After a week of “acclimation” (20 February), the “day” temperature was increased in one room to 30°C, corresponding to a day/night temperature profile of 30°C/15°C, and control vines were retained at 22°C/15°C. Both CE rooms had the same day length of 14 hours, with lights efficiency of 800 μM/m^2^/s turned on from 8 am until 10 pm (**Supplementary Information Figures 1, 2**). The vines were watered regularly to avoid any water stress. Similarly to the first experiment, same process and treatments were applied in the second experiment (2019/20) with the same day length, but the number of potted vines was increased to 30 plants per CE room to allow a replicated time course experiment (measuring berry attributes from pre-veraison to full ripening stage).

### 2.3 Harvesting and sample preparation

On the 13 March 2019 all vines (15 vines/CE rooms) were harvested from the first experiment (2018/19). In the second experiment (2019/20), six randomly selected vines from each CE room were harvested from each of the following time points: pre-veraison 21 February (prior to changing CE room temperature), then at one weekly intervals through the ripening phase (50% veraison 3^rd^, post veraison 10^th^, mid-ripening 17 and harvest 23 March 2020). In both experiments, each vine had one bunch which was harvested and weighed, and the total berries were weighed using a Mettler PE 1600 scale (Watson Victor LTD, Auckland, New Zealand) and placed in 50 mL tubes (~20 berries/tube) before being freeze dried in liquid N_2_ and stored at -80°C freezer. Whole frozen berries from each harvesting time point were ground to powder using an IKA A11 Basic Analytical Mill (Merck KGaA, Darmstadt, Germany). The frozen ground tissue was stored at -80°C prior to further processing for the berry amino acid and biochemistry analysis.

### 2.4 Total soluble solids

Around 1 g of the frozen tissue was left to thaw in 1.5 mL eppendorf tube where berry juice was measured for the total soluble solids (TSS) content expressed as °Brix using a digital refractometer (PR- 100, ATAGO, Japan).

### 2.5 Amino acids analysis

For both experiments, two grams of the frozen grape powder was measured into a 15 mL centrifuge tube, allowed to thaw at room temperature for 20 min. The tubes were vortexed before being centrifuged in Heraeus Multifuge X3R centrifuge (Thermo Fisher Scientific, Auckland, New Zealand) at 3 000 rpm for 5 min. to pellet the solid material. The supernatant was transferred to a new 1.5 mL Eppendorf tube and was diluted 1:4 with distilled water. Each sample was filtered through a 0.25 μm nylon syringe filter (Microlab Scientific Co., Ltd) into an HPLC glass vial and capped tightly. Each sample was analysed using a Hewlett-Packard Agilent 1100 series High Performance Liquid Chromatography (HPLC) system (Waldbronn, Germany) with a 150 mm x 4.6 mm, 3 μM C-18 column (Winlab, Scotland). The pre-column derivatization was performed of Autosampler using o-phthaldialdehyde (OPA) for primary amino acid derivatization reagent, and 9-fluorenylmethyl chloroformate (FMOC) for secondary amino acid derivatization reagent with injection volume of 12.0 µl. Detection utilised a fluorescence detector with an excitation of 335 nm and emission of 440 nm. At 21 min, the detector was switched to a second channel (excitation 260 nm, emission 315 nm) to detect proline. Amino acid standards of known concentrations were analysed in parallel to generate calibration curves for quantification of the unknown samples. The separation used solvent A (0.01 mol/L Na_2_HPO_4_ with 0.8% THF, adjusted to pH 7.5 with H_3_PO_4_) and solvent B (50% methanol, 50% acetonitrile) with the following gradient: 0 min, 0% B; 14 min, 40% B; 20 min, 50% B; 24 min, 100% B; 29 min, 100% B; 30 min, 0% B; 36 min, 0% B; with a flow rate of 0.70 mL/min. The raw data was analysed using the Chemstation chromatography data system (Agilent, CA, USA). Individual amino acids analyzed were alanine, arginine, aspartate, asparagine, cysteine, glutamate, glutamine, glycine, histidine, isoleucine, leucine, lysine, methionine, phenylalanine, proline, serine, threonine, tryptophan, tyrosine, valine. Total amino acid was calculated by summing the concentrations of all detected amino acids.

### 2.6 Phenolic compounds analysis

Total phenolics and anthocyanin compounds were analysed from Pinot noir whole berry ground tissue in the first experiment (2018/19) using an extraction technique that uses acidified 50% aqueous ethanol (Iland et al., 2004). After extraction, the anthocyanin compounds were measured at an absorbance of 520 nm. A measure of total phenolics was obtained by reading the absorbance of the diluted sample at 280 nm. A different method was used in the second experiment (2019/20) which allowed a deeper understanding on the effect of temperature on individual anthocyanins and phenolics. A subsample (1 g) of frozen grape powder was weighed into a 15 mL centrifuge tube and 10 mL of 50% v/v aqueous ethanol (adjusted to pH 2.0 with 1.0 M hydrochloric acid) was added. The tubes were homogenised using a rotary suspension tube mixer (Rateck Instruments Pty Ltd, Victoria, Australia) for 2 hrs before centrifugation at 1 800 g for 10 min. The supernatant was then transferred to a new 15 mL microcentrifuge tube. A portion (1 mL) of the extract was then filtered through a 0.22 μM nylon syringe filter (MicroScience) into an HPLC glass vial. The HPLC analysis of the phenolic compounds and flavonoids was conducted using a Hewlett-Packard Agilent 1100 series (Waldbronn, Germany) following the method used in Gómez-Alonso et al. (2007). Total anthocyanin included the sum of cyanidin-3-glucoside, delphinidin-3-glucoside, malvidin-3-glucoside, petunidin-3-glucoside and peonidin-3-glucoside. The total phenolic and flavonoids included the sum of protocatechuic, caftaric, coumaric, caffeic, gallic acids, procyanidins 1 and 2, catechin, epicatechin, epicatechin gallate, epigallocatechin gallate, quercetin-3-glucoside, quercetin, kaempferol, reveratrol and hydroxyphenyl ethanol. All chemicals and solvents used were purchased from Sigma-Aldrich (Auckland, New Zealand).

### 2.7 Isotopic analysis in berry must

Carbon (δ^13^C) isotope composition were determined in berry juice must for each sample at each harvesting time point for both seasons as described in Gaudillère et al. (2002). Carbon isotope content was measured using a continuous flow isotope ratio mass spectrometer (Sercon Ltd, Crewe, UK) following the method in Avice et al. (1996). Carbon (δ^13^C) is referenced to Vienna- Pee Dee Belemnite (V-PDB).

### 2.8 Statistical analysis

The statistical analyses was conducted using IBM SPSS statistics 26. The effect of temperature on berry weight, TSS, and the accumulation of primary and secondary compounds were determined between the treatments using a one-way analysis of variance (ANOVA) tests between-subjects effects with a Tuckey’s Honest Significant Difference (HSD) at the 5% level. The interaction effect of temperature and time point on berry biochemical data was analysed using a generalized linear model (GLM) with 95% confidence intervals coverage.

## 3 Results

### 3.1 Berry weight and TSS

**Figure 1:**
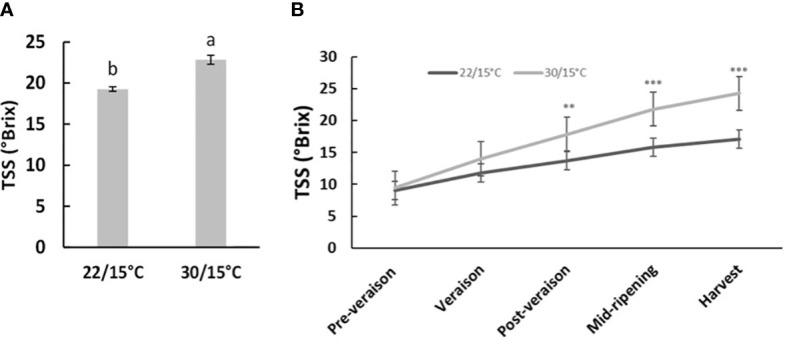
Effect of Temperature treatments (22/15°C and 30/15°C) on Pinot noir total soluble solids (TSS) Brix in the first (2018/19, **(A)** and second experiment (2019/20, **(B)** at harvest and at different time point (2019/20. Graph showing different letter means it is significant at 95% confidence (Tuckey’s HSD test).

The two studied temperatures (22/15°C and 30/15°C) had no significant effect (*p*>0.05) on average berry weight in both experiments at harvest. In the second experiment (2019/2020), berry weight was not affected by the temperature increase at different time points (*p*>0.05, **Supplementary Information, Table 1**). However, TSS at harvest significantly increased by 3.5 and 5.2°Brix in the first- and second-year experimentation respectively for the temperature treatment of 30°C/15°C compared with 22°C/15°C (**Figures 1A, B**). Time course results indicated that TSS at post-veraison and mid-ripening significantly increased by 4.8°Brix and 5.9°Brix respectively for the temperature treatment of 30°C/15°C compared with 22°C/15°C (**Figure 1B**), but no significant differences were observed at pre-veraison and veraison (*p*>0.05, **Supplementary Information, Table 2**).

### 3.2 Total amino acid accumulation in berries

**Figure 2:**
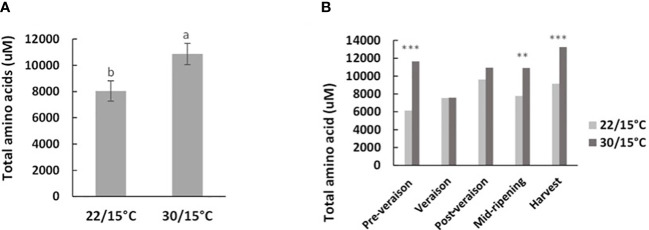
Effect of temperature treatment (22/15°C and 30/15°C) on the accumulation of total amino acids **(A)** and throughout berry development **(B)** in the second experiment (2019/20). Samples are from five time points: pre-veraison, veraison, post-veraison, mid-ripening and ripening. Graph showing different letter means it is significant at 95% confidence (Tuckey’s HSD test). ** p=0.01; *** p=0.001.

Total amino acid concentration at harvest was not significantly influenced by temperature in the first experiment (*p*=0.426). However, in the second experiment, the concentration of amino acids at harvest was significantly higher by 30% in berries grown at 30/15°C compared with berries grown at 22/15°C (*p*=0.002) (**Figure 2A**). The time course in the second experiment indicated that some of these differences were generated earlier on in development: at mid-ripening and harvest stages when the vines had been exposed to the two temperature treatments for 30 and 40 days, total amino concentration in berries increased again for both treatments, but in vines grown at 30/15°C they were significantly higher by 28% and 31% respectively compared with vines grown at 22/15°C (**Figure 2B**). While amino acid berry concentration was 50% higher for the 30/15°C treatment compared with the 22/15°C treatment at pre-veraison, a drastic decrease in amino acid concentration was observed at veraison and there were no differences between treatments (**Figure 2B**). Given that at the veraison timepoint the temperature treatments had only been applied for a few days, it is likely that the pre-veraison differences detected reflect some sampling variability and reflects specific individual amino acid changes.

### 3.3 Individual amino acid accumulation in berries

Of the twenty amino acids analyzed, aspartic acid, arginine, and phenylalanine concentrations significantly decreased by 20%, 35% and 55% respectively and proline, threonine, alanine, and valine concentrations significantly increased by 40%, 15%, 45% and 30% respectively in the 30/15°C treatment compared with the 22/15°C for the first experiment (2018/19) (**Table 1**). In the second experiment (2019/20) most amino acids (except for aspartic acid and phenylalanine) increased at harvest when vines were exposed to 30/15°C compared to 22/15°C temperatures (**Table 1**). Alanine and proline experienced a dramatic increase by 55%, and 65% respectively at 30/15°C compared with 22/15°C. The increase of temperature also resulted in increased concentrations of glutamine and serine by 45% and 40% respectively. The concentration of aspartic acid and phenylalanine were reduced by 20% and 45% respectively at 30/15°C compared with 22/15°C (**Table 1**). The remaining amino acids in both experiments were not significantly influenced by the applied temperature treatments (22/15°C and 30/15°C).

**Table 1:** Effect of temperature treatments (22/15°C and 30/15°C) on the accumulation of individual amino acids at harvest in the first (2018/19) and second experiment (2019/20).

	Experiment (2018/19)			Experiment (2019/20)		
	22/15°C	30/15°C		22/15°C	30/15°C	
Amino Acid	Mean ± SE	Mean ± SE	p value	Mean ± SE	Mean ± SE	p value
α-ketoglutarate						
Glutamine (GLN)	650.45 ± 128.97	606.05 ± 86.18	0.253	462.68 ± 39.14 **b**	848.44 ± 138.88 **a**	0.023
Glutamic acid (GLU)	864.67 ± 46.35	907.09 ± 63.61	0.364	650.52 ± 35.32	818.41 ± 69.87	0.058
Arginine (ARG)	2183.53 ± 363.14 **a**	1406.45 ± 195.17 **b**	0.003	2633.57 ± 447.62	2985.94 ± 610.74	0.652
Proline (PRO)	1228.14 ± 161.77 **b**	2308.44 ± 155.80 **a**	0.001	961.47 ± 284.59 **b**	2516.11 ± 559.29 **a**	0.033
Aspartate						
Aspartic Acid (ASP)	455.61 ± 16.94 **a**	325.86 ± 24.86 **b**	0.002	535.96 ± 45.06 **a**	415.98 ± 25.52 **b**	0.043
Asparagine (ASN)	46.81 ± 5.31	42.64 ± 7.39	0.266	181.74 ± 35.43	132.54 ± 28.65	0.306
Threonine (THR)	879.17 ± 106.69 **b**	703.75 ± 46.62 **a**	0.024	1207.13 ± 92.81	1381.59 ± 112.56	0.259
Isoleucine (ILE)	77.74 ± 11.16	74.75 ± 10.18	0.132	147.11 ± 10.90	147.11 ± 15.99	0.744
Methionine (MET)	42.57 ± 5.39	43.36 ± 3.47	0.665	45.02 ± 4.18	60.36 ± 9.14	0.158
Lysine (LYS)	51.45 ± 2.76	48.06 ± 2.32	0.436	39.91 ± 5.51	53.36 ± 9.06	0.233
Pyruvate						
Leucine (LEU)	128.55 ± 17.40	106.64 ± 11.52	0.365	207.36 ± 16.23	222.72 ± 30.15	0.663
Alanine (ALA)	549.55 ± 74.61 **b**	929.96 ± 102.92 **a**	0.004	989.50 ± 208.74 **b**	2032.39 ± 400.91 **a**	0.044
Valine (VAL)	123.51 ± 15.16 **b**	173.09 ± 15.81 **a**	0.035	155.94 ± 22.55	277.16 ± 54.21	0.066
Aromatic						
Phenylalanine (PHE)	65.54 ± 8.74 **a**	35.31 ± 2.28 **b**	0.001	65.09 ± 7.95	58.39 ± 3.49	0.458
Tryptophan (TRY)	87.85 ± 5.49	85.18 ± 3.47	0.469	127.61 ± 12.01	128.07 ± 4.58	0.972
Tyrosine (TYR)	32.68 ± 5.50	37.25 ± 3.52	0.478	53.65 ± 6.63	71.78 ± 11.49	0.202
Histidine (HIS)	269.21 ± 13.57	250.32 ± 13.48	0.625	107.81 ± 14.73	133.33 ± 17.46	0.290
3-phosphoglycerate						
Serine (SER)	373.28 ± 50.35	417.41 ± 28.92	0.315	440.37 ± 31.63 **b**	778.65 ± 115.34 **a**	0.018
Glycine (GLY)	70.78 ± 4.09	70.21 ± 3.69	0.453	52.11 ± 3.95	72.94 ± 8.69	0.054
Cysteine (CYS)	103.87 ± 4.24	101.47 ± 8.17	0.564	106.14 ± 7.81	122.20 ± 9.06	0.209

Amino acids mean ± SE showing different bold letter means it is significant at 95% confidence (Tuckey’s HSD test).

**Figure 3:**
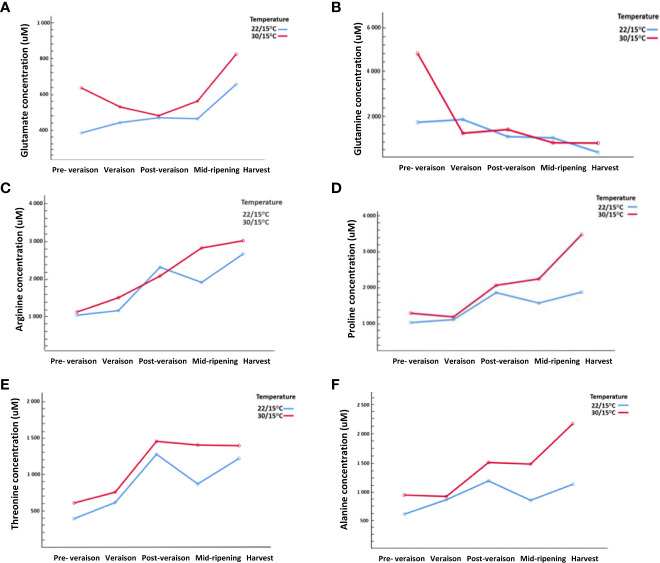
Effect of temperature (22/15°C and 30/15°C) on specific amino acids concentrations (uM) in Pinot noir throughout berry development (pre-veraison, veraison, port-veraison, mid-ripening and ripening) in 2019/20. **(A)** Glutamate, **(B)** Glutamine, **(C)** Arginine, **(D)** Proline, **(E)** Alanine, **(F)** Threonine.

The second experiment (2019/20) provided insights into the origins of changes in amino acid concentrations measured at harvest through the sampling of vines at early developmental times (**Supplementary Information Table 3**). The concentration of glutamate was high at pre- veraison at 30/15°C followed by a decrease at post-veraison but then increased in concentration from mid-ripening to harvest. Contrastingly, at 22/15°C, glutamate continuously increased throughout berry ripening (**Figure 3A**). Similarly, glutamine concentration at pre-veraison was higher at 30/15°C compared with 22/15°C but then decline with berry ripening in response to both temperatures (**Figure 3B**). Other amino acids such as arginine, proline, alanine, and threonine showed a noticeable post-veraison peak at both temperatures (**Figure 3**). The accumulation of arginine began even before veraison and increased continuously during ripening. At 30/15°C, arginine showed a linear accumulation, however, at 22/15°C a significant increase was observed post-veraison followed by a decrease mid-ripening and then an increase at ripening (**Figure 3C**). The concentration of proline significantly increased post-veraison with a significant increase in proline concentration from mid-ripening to harvest at 30/15°C compared with 22/15°C. (**Figure 3D**). Alanine and threonine were also influenced by the temperature, with both amino acids having higher concentrations at 30/15°C compared with 22/15°C at post-veraison and harvest (**Figures 3E, F**). Despite mostly no differences at harvest (**Table 1**), other amino acids (i.e., cysteine, histidine, valine, methionine, tryptophane and lysine) with very low concentrations were also significantly influenced (*p*<0.05) by the time point and temperature interaction with a clear increase in concentration at 30/15°C compared with 22/15°C throughout berry development (**Supplementary Information Table 3**).

### 3.4 Total anthocyanins and phenolics

**Figure 4:**
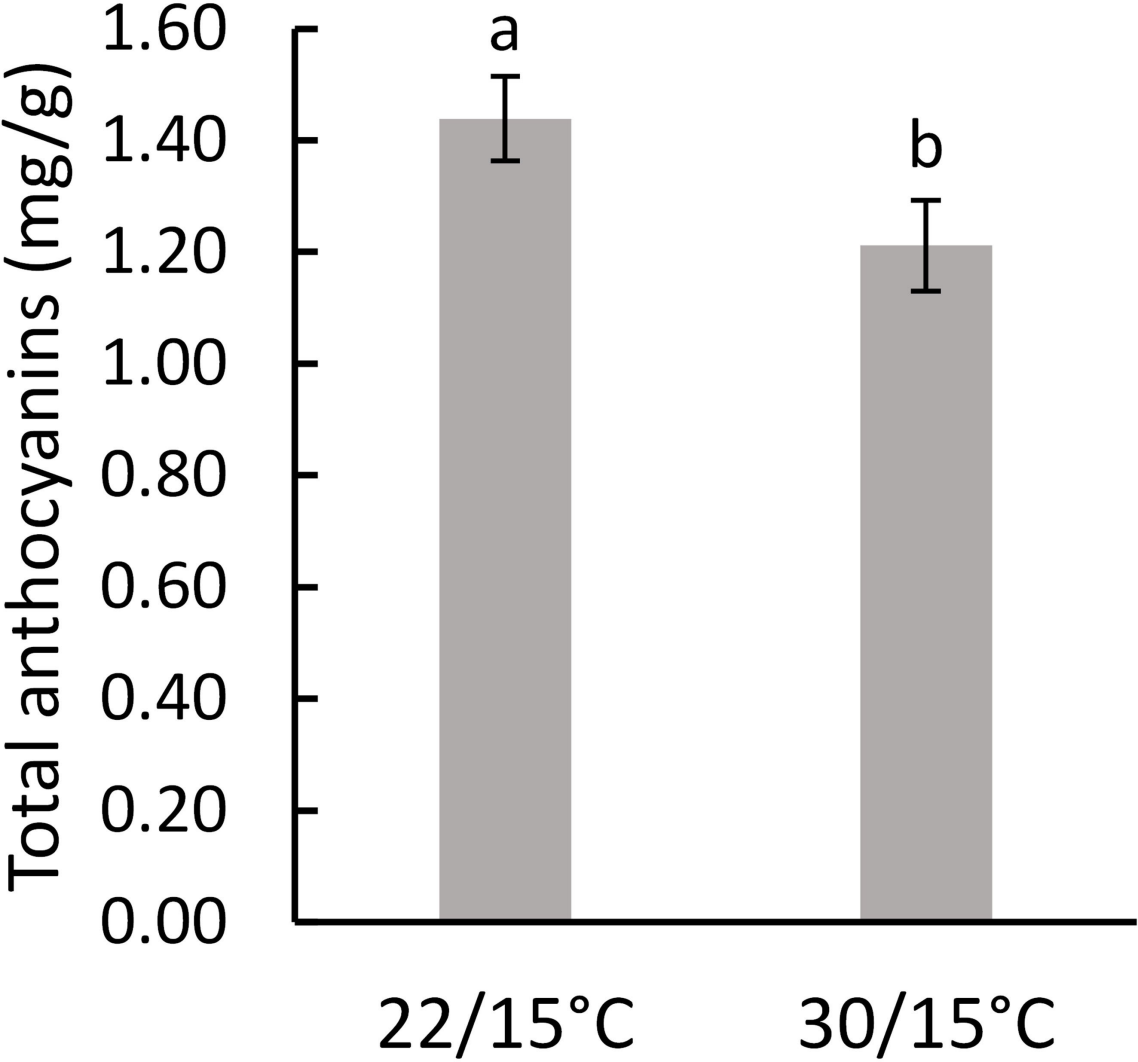
Effect of temperature (22/15°C and 30/15°C) on total anthocyanins in Pinot noir grapes at harvest in the first experiment (2018/19). Graph showing different letter means it is significant at 95% confidence (Tuckey’s HSD test).

In the first experiment (2018/19), there were no significant differences (*p*=0.654) between the two temperatures on total phenolic concentrations as measured at harvest (**Table 2**). A significant decrease (*p*=0.023) in anthocyanins content (by 15%) was observed in grape berries in vines grown at 30/15°C compared with 22/15°C (**Figure 4**). In the second experiment (2019/2020), total phenolics and total anthocyanins were not significantly influenced (*p*=0.227 and *p*=0.705, respectively) by the temperature treatments (22/15°C and 30/15°C) (**Table 2**).

**Table 2:** Effect of temperature (22/15°C and 30/15°C) on the concentration of total phenolics and total anthocyanins in Pinot noir grapes at harvest in the first (2018/2019) and second experiment (2019/20).

	22/15°C		30/15°C		
	Mean (mg/g)	SE	Mean	SE	p value
	2018/2019				
Total phenolics	1.73	0.06	1.78	0.08	0.654
Total Anthocyanins	1.44 **a**	0.08	1.21 **b**	0.08	0.023
	2019/2020				
Total phenolics	1.99	0.34	1.50	0.18	0.227
Total Anthocyanins	0.78	0.10	0.74	0.05	0.705

Different bold letter means it is significant at 95% confidence (Tuckey’s HSD test).

### 3.5 Individual phenolics and anthocyanin accumulation in berries (Experiment 2019/20 only)

Temperature had no significant effect on individual phenolics and individual anthocyanins (*p*>0.05) (**Table 3**). However, specific phenolic compounds such as epicatechin, procyanidin, caftaric acid and caffeic acid were significantly influenced (*p*<0.05) by the interaction between the temperature and the developmental time (**Supplementary Information Tables 4, 5**). For these phenolic compounds further investigation was conducted to look at the effect of temperature at each time point (**Supplementary Information Table 6**); these compounds had one or two significant differences with greater concentrations in the 30/15°C treatment, however, these results were not consistent across the full-time course.

**Table 3:** Effect of temperature (22/15°C and 30/15°C) on the concentration of individual phenolics in Pinot noir grapes at harvest in the second experiment (2019/20).

	Temperature	Mean (mg/g)	Std. Error	95% Confidence Interval (CI)			p value
				Lower Bound	Upper Bound		
Individual phenolics							
Catechin	22/15°C	127.48	24.08	73.82	181.13		0.254
	30/15°C	86.24	24.08	32.58	139.89		
Epigallocatechin	22/15°C	30.91	3.49	23.13	38.69		0.420
	30/15°C	26.76	3.49	18.98	34.54		
Epicatechin	22/15°C	16.99	5.26	5.26	28.72		0.725
	30/15°C	14.30	5.26	2.57	26.02		
Procyanidin B1	22/15°C	6.53	0.89	4.53	8.52		0.713
	30/15°C	7.01	0.89	5.01	9.00		
Resveratrol	22/15°C	6.42	0.54	5.22	7.61		0.099
	30/15°C	5.04	0.54	3.85	6.23		
Caftaric acid	22/15°C	3.80	0.43	2.83	4.77		0.945
	30/15°C	3.76	0.43	2.79	4.72		
Procyanidin B2	22/15°C	2.86	0.48	1.78	3.93		0.971
	30/15°C	2.83	0.48	1.76	3.90		
Quercetin	22/15°C	2.77	0.40	1.87	3.66		0.661
	30/15°C	2.51	0.40	1.62	3.41		
Gallic acid	22/15°C	0.85	0.15	0.51	1.19		0.756
	30/15°C	0.78	0.15	0.44	1.12		
Caffeic acid	22/15°C	0.46	0.05	0.35	0.56		0.810
	30/15°C	0.48	0.05	0.37	0.58		
Epicatechin gallate	22/15°C	0.38	0.04	0.29	0.46		0.348
	30/15°C	0.33	0.04	0.24	0.41		
Individual anthocyanins							
Malvidin	22/15°C	15.84	1.67	12.11	19.57		0.527
	30/15°C	14.29	1.67	10.56	18.02	
Peonidin	22/15°C	5.77	1.84	4.02	7.50		0.816
	30/15°C	5.55	1.84	3.75	7.25		
Petunidin	22/15°C	4.37	1.77	0.43	8.32		0.785
	30/15°C	3.67	1.77	-0.28	7.62		
Delphinidin	22/15°C	0.53	0.10	0.29	0.76		0.055
	30/15°C	0.84	0.10	0.61	1.07		

CI mean confidence interval at 95%.

### 3.6 Carbon isotope analysis

Carbon isotope (δ^13^C) composition in the berry must at harvest was not significantly different (*p*>0.05) between the two studied temperature treatments (22°C/15°C and 30°C/15°C) (**Table 4**).

**Table 4:** Effect of temperature (22/15°C and 30/15°C) on Carbon isotope (δ^13^C) composition in the berry must in the first (2018/20) and second (2019/20) experiment.

Temperature		2018/20	2019/20
22°C/15°C	Mean	-29.51	-28.72
	SE	0.25	0.31
30°C/15°C	Mean	-29.36	-28.46
	SE	0.34	0.27
*p* value		0.451	0.258

## 4 Discussion

Modifying the bunch microclimate through increasing temperatures at veraison on Pinot noir grapevine cultivar provided a means to explore the impacts of climate change on the accumulation of primary and secondary metabolites through ripening and at harvest. Of note, this is the first study to provide a detailed analysis and quantification of individual amino acids and phenolics in Pinot noir in response to changes in temperature applied at veraison.

Average berry weight at harvest was not influenced by temperature treatments in both experiments which confirms findings from another study where temperatures of 20 and 30°C had no effect on berry weight in red grapevine cultivar “Aki Queen” from fruit set to maturity (Yamane et al., 2006). This could indicate that berry growth was not affected by increasing temperatures with the chosen temperatures or the timing of application. The no change in berry weight with the studied temperature could also be because water stress was not a parameter assessed and the vines were irrigated. Other studies showed that an inhibition or cessation of berry growth was only observed when temperature was above 35°C at veraison and mid-ripening (Greer and Weston, 2010; Greer and Weedon, 2013). Moreover, total soluble solids were increased in temperature treatment of 30°C/15°C compared with 22°C/15°C, in agreement with previous reports where TSS accumulation was stimulated by high temperatures 30°C and above applied at veraison or a few days after (Mori et al., 2005; Poudel et al., 2009; Lecourieux et al., 2017). However, other studies showed that when high temperatures above 30°C were applied from fruit set to maturity, TSS was not influenced by temperature or heating treatments in other red grape varieties (Yamane et al., 2006; Sadras et al., 2013; Wu et al., 2019). Our results suggest that higher temperatures from veraison onwards might influence berry development resulting in early ripening of Pinot noir. This is crucial for Pinot noir wine regions in New Zealand and globally as such elevated temperatures are predicted to be common in future climate change scenarios. Earlier harvest dates due to climate change have been reported when temperatures increased significantly during the ripening period (Cook and Wolkovich, 2016; van Leeuwen and Darriet, 2016; García de Cortázar-Atauri et al., 2017; van Leeuwen and Destrac, 2017; Labbé, 2019). Even though this study did not look at the effect of temperature on titratable acidity, studies suggest that the increase of temperature above 30°C alter and reduce the titratable acidity in several grape varieties during berry development (Greer and Weston, 2010; Arrizabalaga-Arriazu et al., 2020; Venios et al., 2020; Deloire et al., 2021; Xyrafis et al., 2022).

Individual amino acids detected in Pinot noir berries in this study were similar to other studies (Stines et al., 2000; Lee and Schreiner, 2010; Schreiner et al., 2013). These studies also reported the presence of other amino acids such as gamma-amino butyric acid, ornithine in Stines et al. (2000), hydroxyproline and citrulline in Lee and Schreiner (2010) and Schreiner et al. (2013) but these amino acids were not found in the current study. Other amino acids such as cysteine were not detected in Pinot noir berries in any of these studies and the presence of tryptophan was not detected in Stines et al. (2000) but were detected at lower concentrations in this study. The differences in detection of certain amino acids could be related to the different extraction methods or standards tested. For example, gamma-amino butyric acid was not detected in the current study due to the absence of the respective standard. Moreover, the introduction of varied dilution factor in the extraction step in this study could have contributed to the absence of hydroxyproline and citrulline as also explained in Stines et al. (2000). Threonine and alanine concentrations were relatively high in Pinot noir berries for both temperature treatments. Stines et al. (2000) found that these two amino acids are characteristically higher in Pinot noir berries compared with other grape varieties. Irrespective of temperatures, the decline of glutamine concentration during grape berry development indicates that glutamine is converted to other amino acids (such as proline and arginine) as suggested in other studies (Stines et al., 1999; Stines et al., 2000).

The increase of temperature led to a higher accumulation of most individual amino acids in the second experiment (2019/20). This is in line with other studies which have observed a significant increase in seven amino acids (threonine, arginine, tyrosine, phenylalanine, cysteine, lysine and γ-aminobutyric acid) in greenhouse-grown grape at higher temperature (35°C) applied to the bunch zone during veraison, or ripening stages (Lecourieux et al., 2017). However, in this current study, these reported amino acids were not affected by temperature except for threonine. Moreover, another study showed that the application of elevated temperature (35/20°C) that last 11 days applied at veraison has positively affected free amino acids (valine, leucine, serine, glycine, aspartate, threonine, isoleucine, glutamate, proline, and γ-aminobutyric acid) in Shiraz berries (Sweetman et al., 2014). These amino acids (except for γ-aminobutyric acid, leucine, glycine, and isoleucine) were increased in current study with higher temperature treatment (30/15°C). The increase of proline and arginine were observed at high concentrations under the higher temperature treatment, and this has also been suggested in other studies (Lecourieux et al., 2017; Torres et al., 2017). In the later stages of the ripening process, proline increase could be linked to the increased sugar accumulation at higher temperature during this period (Jogaiah et al., 2010). This increase could be related to proline having a protective role in plants against abiotic stresses, including elevated temperature (Garde-Cerdán et al., 2011). This is consistent with other studies where the concentrations of proline and arginine increased in response to higher temperatures such as 28°C (Torres et al., 2017) and 35°C (Lecourieux et al., 2017). The contrasting changes in glutamate concentration between the two temperature treatments (22°C/15°C and 30°C/15°C) in this study could indicate a stress response to the elevated temperature consistent with an increase in transcript level of the heat shock protein gene seen in these berries at veraison under field conditions (Ford, 2015). According to Obata and Fernie (2012), threonine accumulated under abiotic stress conditions which in this study could be associated with the higher temperature (30/15°C) treatment. Another study suggested that threonine concentration accumulate under drought stress conditions (Joshi et al., 2010), however, this was not the case in this study as the isotope ^13^C in the berries was not significant between the two studied temperature treatments (22°C/15°C and 30°C/15°C) and the vines were watered as required indicating no water stress.

Contrasting results in the accumulation of specific amino acids between the two seasons were that arginine, threonine, and phenylalanine concentrations were significantly decreased at 30/15°C in the first experiment (2018/19) but they did not significantly change in the second experiment (2019/20). The difference in certain amino acid profile in both seasons could be related to cultivars, rootstock/scion combinations, vine nutrient management, and growing season as reported by other authors (Huang and Ough, 1989; Gump et al., 2002; Bell and Henscken, 2005). In this study, the difference in arginine could be due to the change in leaf system in the clone variation (own rooted PN *vs.* Abel/3309 PN) where the transport of amine occurs. As leaf area measurements were not taken in this study, further investigation of this hypothesis would require quantification to elucidate whether this may be playing a role. Moreover, studies have highlighted the impact of rootstocks on amino acid profile. It was shown that the rootstock genotype altered the concentration of most amino acids such as arginine, glutamine, proline, and histidine in Pinot noir (Blank et al., 2022). In another study, Shiraz grafted onto a Schwarzmann rootstock had the highest juice free amino acid concentration of all Shiraz/rootstock treatment wines (Treeby et al., 2000). This can also be related to the nitrogen availability and its assimilation in different rootstocks (Bell and Henscken, 2005).

The increased accumulation of certain amino acids in this study can have a positive effect on the YAN which is a good indication of high nitrogen availability and less chance of stuck ferment. High amino acid concentration may have a good impact on the quality control in sense off flavors compounds can be avoided (yeasts are not stressed). In addition, wine growers/makers would require less input of organic and inorganic nitrogen. As for wine quality, having high specific amino acids at early stages of the fermentation can produce wines with good complexity in terms texture and mouthfeel (glycerol etc.). It was recently reported that the presence of proline at high concentration can provide certain benefits to the wines such as increased sweetness, viscosity and flavor and lower astringency and bitterness (Nandorfy et al., 2022). Therefore, studies need to investigate the effect of microbes on proline under natural fermentation conditions. In addition, Amino acids are the starting precursors for important secondary compounds in grapes such as phenolic compound, methoxypyrazines, thiols, esters, flavonols and anthocyanins etc. Many other compounds that play an important role in determining the flavour and aroma profile of a wine (e.g., thiols) need biochemical modifications by yeasts during fermentation. This means that any change in amino acid composition can influence yeast growth and fermentation and ultimately impact wine quality and secondary composition (Gregan, 2019). In the current study, threonine increase at high temperature could potentially lead in higher secondary alcohols, acids, and esters in the wine. Hernandez-Orte et al. (2002) found that threonine and phenylalanine to have the most influence on wine aroma composition. Other amino acids such as valine was also increased in concentration with temperature increase and this might cause an increase in methoxypyrazines which is found in high concentration in New Zealand Sauvignon blanc (Tsai et al., 2022). Studies have suggested that other amino acids have also been correlated to differences in wine quality components (Rapp and Versini, 1996; Hernandez-Orte et al., 2002; Bell and Henscken, 2005). Moreover, future research needs to follow these changes currently measure in the berries in wine making and sensory.

Anthocyanins are compounds synthesized *via* the phenylpropanoid pathway through phenylalanine (He et al., 2010). Therefore, the substantial decrease of phenylalanine at 30/15°C in the first experiment could be associated with the decrease of anthocyanins in the berries as seen in other studies where high temperatures decrease the expression of genes related to flavonoid biosynthesis in grape skins and increase anthocyanin degradation (Mori et al., 2005; Mori et al., 2007). Total anthocyanin reduction in response to elevated temperature in this study was also observed in other studies where anthocyanins were reduced in response to higher temperatures of 30°C (Yamane et al., 2006) and above 35°C (Azuma et al., 2012; Koshita et al., 2015; Shinomiya et al., 2015; Ryu et al., 2020).

This study showed that total and individual phenolic at harvest were not affected by temperature treatments. This came in confirmation with other studies where phenolic compounds such as tannins and flavan-3-ols were less affected by temperature or heat stress (Spayd et al., 2002; Cohen et al., 2008; Pastore et al., 2017; Gouot et al., 2019). Furthermore, it is reported that phenolic compounds (i.e., flavonoids) are mostly responsive to light whose levels increased with better sunlight exposure (Song et al., 2015; Xi et al., 2016; Brandt et al., 2019; Blancquaert et al., 2019). It is suggested that light and particularly UV-B has been shown to have a substantial effect on flavonoids (Gregan et al., 2012; Liu et al., 2015), so future studies of light on Pinot noir is appropriate. Epicatechin is the basic monomeric unit of grape and wine tannins, and significant reduction of this compound may lead to the decrease of condensed tannins, which may consequently reduce the perception of astringency and bitterness (Watrelot and Norton, 2020). Procyanidin B1 content decreased at higher temperature at veraison and mid-ripening stage. Procyanidin B1 is a procyanidins dimer of epicatechin and catechin, which also contributes to the perception of astringency (Hornedo-Ortega et al., 2021). In a potted vine system, procyanidins content in grape berry at 30°C in a phytotron during veraison were significantly reduced (Poudel et al., 2009).

## 5 Conclusions

Complex number of environmental and biological factors will determine the concentration of metabolites in grape berries and these factors will vary under a range of different circumstances. The bunch microclimate-controlled experiments demonstrated that the increase of temperature to 30/15°C enhance the accumulation of TSS in the berries in both experiments. Temperature increase did not affect berry weight or cause water loss as grapevines were grown under an irrigated experiment design and water was not a limiting factor. Temperature influenced the accumulation of amino acids specifically the α-ketoglutarate family (i.e., glutamine, arginine, and proline). Proline accumulation increased with the higher temperature treatment. Other amino acids with lower concentration (i.e., aspartic acid and serine) also increased with higher temperature throughout berry development especially in the second experiment. These observations could show a potential source of wine typicity changes in relationship with climate change since amino acid profiles may be closely linked to the aromatic profiles of wines. In addition, the increase of temperature had a negative effect on anthocyanin content in the berry in one of the two experiments, which could also play a detrimental role in wine qualities under warming climates. While total phenolics were not influenced by temperature, further studies could investigate light and particularly UV-B (so cofounding elements with temperature) on Pinot noir total phenolic characteristics. Moreover, future experiments are required to assess phenolics and amino acid accumulations under high temperature with simulated water stress as an added factor causing nitrogen limitation in grapevines due to limited water availability.

## Data availability statement

The original contributions presented in the study are included in the article/**Supplementary Material**. Further inquiries can be directed to the corresponding author.

## Author contributions

BJ and SG contributed to conception and design of the study. SG set up the experiments. RM and SG collected and organized the data. RM performed the statistical analysis and RM, BJ, AP, and OS contributed to interpretation of the statistical analysis. RM wrote the first draft of the manuscript. BJ, AM and OS wrote sections of the manuscript. All authors contributed to manuscript revision, read, and approved the submitted version.
