# Supplementary material for: Bunch microclimate influence amino acids and phenolic profiles of Pinot noir grape berries
Source: Front Plant Sci. 2023 Jun 7;14:1162062. doi: 10.3389/fpls.2023.1162062 (PMC10282841; doi:10.3389/fpls.2023.1162062)
Supplement: Supplementary file 1 [file DataSheet_1.docx]

Supplementary Material

**Bunch microclimate influence amino acids and phenolic profiles of Pinot noir grape berries**

**Romy Moukarzel^1^*, Amber K. Parker^1^, Olaf J. Schelezki^1^, Scott M. Gregan^2^, and Brian Jordan^1^**

^1^ Department of Wine, Food and Molecular Biosciences, Lincoln University, Lincoln 7647, New Zealand

^2^ Ministry of Primary Industries, Burnside, Christchurch 8053, New Zealand

# * Correspondence: Romy Moukarzel [romy.moukarzel@lincoln.ac.nz](mailto:romy.moukarzel@lincoln.ac.nz)

# Supplementary Figures and Tables

## Supplementary Figures

The figures below show the light and temperature monitored in both EC chambers.

**
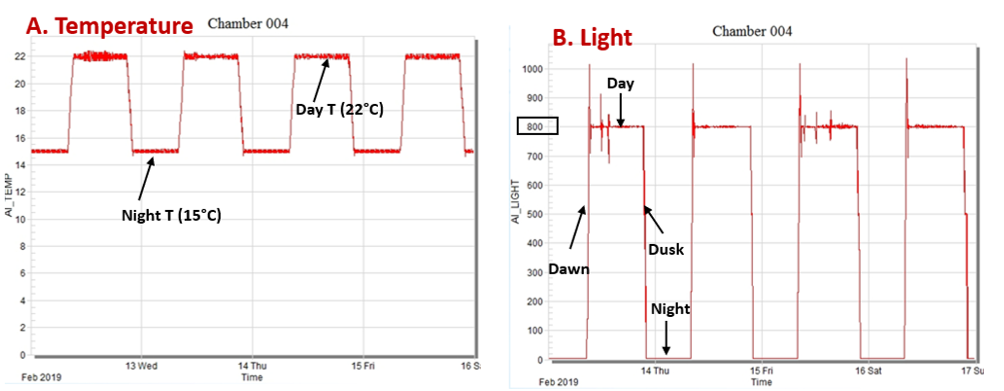
**

**Supplementary Figure 1.** Controlled environment (CE) indicating A. Diurnal change in day and night 22°C/15°C and B. light controlled at 800 μM/m^2^/s.

**
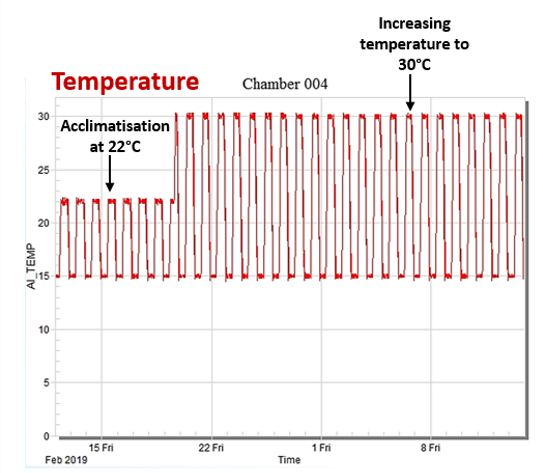
**

**Supplementary Figure 2.** Controlled environment (CE) indicating the week acclimatization at 22°C/15°C before increasing temperature to 30°C/15°C.

Supplementary Table 1*.* Effect of temperature (22/15°C and 30/15°C) on berry weight at different time point in the second experiment (2019/20). CI mean confidence interval at 95%.

|  |  |  |  | 95% Confidence Interval | |  |
| --- | --- | --- | --- | --- | --- | --- |
| Time point | Temperature | Berry weight (g) | Std. Error | Lower Bound | Upper Bound | p value |
| Pre- veraison | 22/15°C | 0.84 | 0.09 | 0.66 | 1.03 | 0.624 |
|  | 30/15°C | 0.97 | 0.09 | 0.79 | 1.16 |  |
| Veraison | 22/15°C | 1.09 | 0.09 | 0.90 | 1.27 | 0.742 |
|  | 30/15°C | 1.16 | 0.11 | 0.93 | 1.39 |  |
| Post-veraison | 22/15°C | 1.33 | 0.09 | 1.14 | 1.51 | 0.612 |
|  | 30/15°C | 1.18 | 0.09 | 0.99 | 1.36 |  |
| Mid-ripening | 22/15°C | 1.58 | 0.09 | 1.39 | 1.77 | 0.751 |
|  | 30/15°C | 1.53 | 0.09 | 1.34 | 1.71 |  |
| Ripening | 22/15°C | 1.62 | 0.09 | 1.43 | 1.81 | 0.568 |
|  | 30/15°C | 1.43 | 0.09 | 1.24 | 1.61 |  |

Supplementary Table 2*.* Effect of temperature (22/15°C and 30/15°C) on TSS (°Brix) at different time point in the second experiment (2019/20). CI mean confidence interval at 95%.

|  |  |  |  | 95% Confidence Interval | |  |
| --- | --- | --- | --- | --- | --- | --- |
| Time point | Temperature | TSS (°Brix) | Std. Error | Lower Bound | Upper Bound | p value |
| Pre- veraison | 22/15°C | 10.07 | 0.40 | 9.25 | 10.88 | 0.423 |
|  | 30/15°C | 9.45 | 0.40 | 8.64 | 10.26 |  |
| Veraison | 22/15°C | 11.82 | 0.40 | 11.00 | 12.63 | 0.075 |
|  | 30/15°C | 14.03 | 0.49 | 13.03 | 15.02 |  |
| Post-veraison | 22/15°C | 13.67 | 0.40 | 12.85 | 14.48 | 0.024 |
|  | 30/15°C | 17.88 | 0.40 | 17.07 | 18.70 |  |
| Mid-ripening | 22/15°C | 15.83 | 0.40 | 15.02 | 16.65 | 0.001 |
|  | 30/15°C | 21.78 | 0.40 | 20.97 | 22.60 |  |
| Ripening | 22/15°C | 17.07 | 0.40 | 16.25 | 17.88 | 0.001 |
|  | 30/15°C | 24.27 | 0.40 | 23.45 | 25.08 |  |

Supplementary Table 3. Effect of temperature (22/15°C and 30/15°C) on the concentration of individual amino acids in Pinot noir grapes at different time point in the second experiment (2019/20). CI mean confidence interval at 95%.

| Dependent Variable |  |  | Mean | Std. Error | 95% Confidence Interval | |  |
| --- | --- | --- | --- | --- | --- | --- | --- |
| Amino acid | Time point | Temperature | (µM) |  | Lower Bound | Upper Bound | *p* value |
| Aspartic acid | Pre-veraison | 22°C | 274.47 | 41.48 | 191.07 | 357.87 | 0.259 |
|  |  | 30°C | 488.73 | 41.48 | 405.33 | 572.12 |  |
|  | Veraison | 22°C | 360.04 | 41.48 | 276.64 | 443.44 |  |
|  |  | 30°C | 402.95 | 50.80 | 300.81 | 505.10 |  |
|  | Post-veraison | 22°C | 301.65 | 41.48 | 218.25 | 385.04 |  |
|  |  | 30°C | 273.23 | 41.48 | 189.83 | 356.63 |  |
|  | Mid-ripening | 22°C | 395.53 | 41.48 | 312.13 | 478.93 |  |
|  |  | 30°C | 440.44 | 41.48 | 357.04 | 523.84 |  |
|  | Ripening | 22°C | 535.97 | 41.48 | 452.57 | 619.37 |  |
|  |  | 30°C | 415.99 | 41.48 | 332.59 | 499.39 |  |
| Glutamate | Pre-veraison | 22°C | 379.54 | 55.00 | 268.96 | 490.13 | 0.001 |
|  |  | 30°C | 630.49 | 55.00 | 519.90 | 741.07 |  |
|  | Veraison | 22°C | 437.01 | 55.00 | 326.42 | 547.59 |  |
|  |  | 30°C | 524.47 | 67.36 | 389.03 | 659.90 |  |
|  | Post-veraison | 22°C | 464.03 | 55.00 | 353.44 | 574.61 |  |
|  |  | 30°C | 475.62 | 55.00 | 365.04 | 586.21 |  |
|  | Mid-ripening | 22°C | 458.06 | 55.00 | 347.47 | 568.64 |  |
|  |  | 30°C | 556.50 | 55.00 | 445.91 | 667.08 |  |
|  | Ripening | 22°C | 650.52 | 55.00 | 539.93 | 761.10 |  |
|  |  | 30°C | 818.41 | 55.00 | 707.83 | 929.00 |  |
| Cysteine | Pre-veraison | 22°C | 178.64 | 23.32 | 131.76 | 225.52 | 0.044 |
|  |  | 30°C | 265.03 | 23.32 | 218.15 | 311.91 |  |
|  | Veraison | 22°C | 163.62 | 23.32 | 116.74 | 210.49 |  |
|  |  | 30°C | 174.45 | 28.55 | 117.04 | 231.86 |  |
|  | Post-veraison | 22°C | 134.03 | 23.32 | 87.15 | 180.91 |  |
|  |  | 30°C | 148.69 | 23.32 | 101.82 | 195.57 |  |
|  | Mid-ripening | 22°C | 180.95 | 23.32 | 134.07 | 227.83 |  |
|  |  | 30°C | 209.36 | 23.32 | 162.48 | 256.24 |  |
|  | Ripening | 22°C | 106.15 | 23.32 | 59.27 | 153.02 |  |
|  |  | 30°C | 122.19 | 23.32 | 75.31 | 169.07 |  |

… AA Table continues

|  |  |  | Mean | Std. Error | 95% Confidence Interval | |  |
| --- | --- | --- | --- | --- | --- | --- | --- |
| Amino acid | Time point | Temperature | (µM) |  | Lower Bound | Upper Bound | *p* value |
| Asparagine | Pre-veraison | 22°C | 165.58 | 47.75 | 69.57 | 261.60 | 0.389 |
|  |  | 30°C | 280.25 | 47.75 | 184.23 | 376.26 |  |
|  | Veraison | 22°C | 167.03 | 47.75 | 71.02 | 263.05 |  |
|  |  | 30°C | 215.39 | 58.49 | 97.79 | 332.98 |  |
|  | Post-veraison | 22°C | 207.40 | 47.75 | 111.38 | 303.41 |  |
|  |  | 30°C | 205.55 | 47.75 | 109.53 | 301.56 |  |
|  | Mid-ripening | 22°C | 187.99 | 47.75 | 91.97 | 284.00 |  |
|  |  | 30°C | 210.64 | 47.75 | 114.62 | 306.65 |  |
|  | Ripening | 22°C | 181.74 | 47.75 | 85.72 | 277.75 |  |
|  |  | 30°C | 132.54 | 47.75 | 36.52 | 228.55 |  |
| Serine | Pre-veraison | 22°C | 538.46 | 111.52 | 314.24 | 762.68 | 0.083 |
|  |  | 30°C | 722.23 | 111.52 | 498.01 | 946.45 |  |
|  | Veraison | 22°C | 878.82 | 111.52 | 654.60 | 1103.04 |  |
|  |  | 30°C | 501.26 | 136.58 | 226.65 | 775.88 |  |
|  | Post-veraison | 22°C | 755.29 | 111.52 | 531.06 | 979.51 |  |
|  |  | 30°C | 946.50 | 111.52 | 722.28 | 1170.72 |  |
|  | Mid-ripening | 22°C | 352.48 | 111.52 | 128.26 | 576.70 |  |
|  |  | 30°C | 655.60 | 111.52 | 431.38 | 879.82 |  |
|  | Ripening | 22°C | 440.38 | 111.52 | 216.15 | 664.60 |  |
|  |  | 30°C | 778.65 | 111.52 | 554.43 | 1002.87 |  |
| Glutamine | Pre-veraison | 22°C | 1717.98 | 701.07 | 308.39 | 3127.57 | 0.229 |
|  |  | 30°C | 4586.13 | 701.07 | 3176.54 | 5995.72 |  |
|  | Veraison | 22°C | 1834.93 | 701.07 | 425.34 | 3244.52 |  |
|  |  | 30°C | 1262.21 | 858.63 | -464.18 | 2988.59 |  |
|  | Post-veraison | 22°C | 1119.22 | 701.07 | -290.37 | 2528.81 |  |
|  |  | 30°C | 1413.01 | 701.07 | 3.42 | 2822.60 |  |
|  | Mid-ripening | 22°C | 1068.98 | 701.07 | -340.61 | 2478.57 |  |
|  |  | 30°C | 861.98 | 701.07 | -547.61 | 2271.57 |  |
|  | Ripening | 22°C | 462.68 | 701.07 | -946.91 | 1872.27 |  |
|  |  | 30°C | 848.45 | 701.07 | -561.14 | 2258.03 |  |

… AA Table continues

|  |  |  | Mean | Std. Error | 95% Confidence Interval | |  |
| --- | --- | --- | --- | --- | --- | --- | --- |
| Amino acid | Time point | Temperature | (µM) |  | Lower Bound | Upper Bound | *p* value |
| Histidine | Pre-veraison | 22°C | 116.86 | 22.54 | 71.55 | 162.18 | 0.02 |
|  |  | 30°C | 179.45 | 22.54 | 134.13 | 224.76 |  |
|  | Veraison | 22°C | 148.25 | 22.54 | 102.93 | 193.56 |  |
|  |  | 30°C | 200.41 | 27.60 | 144.91 | 255.90 |  |
|  | Post-veraison | 22°C | 155.77 | 22.54 | 110.45 | 201.08 |  |
|  |  | 30°C | 204.01 | 22.54 | 158.70 | 249.32 |  |
|  | Mid-ripening | 22°C | 149.07 | 22.54 | 103.75 | 194.38 |  |
|  |  | 30°C | 136.81 | 22.54 | 91.50 | 182.13 |  |
|  | Ripening | 22°C | 107.81 | 22.54 | 62.50 | 153.13 |  |
|  |  | 30°C | 133.34 | 22.54 | 88.02 | 178.65 |  |
| Glycine | Pre-veraison | 22°C | 104.70 | 11.37 | 81.85 | 127.55 | 0.402 |
|  |  | 30°C | 120.91 | 11.37 | 98.06 | 143.76 |  |
|  | Veraison | 22°C | 119.12 | 11.37 | 96.27 | 141.97 |  |
|  |  | 30°C | 92.33 | 13.92 | 64.34 | 120.31 |  |
|  | Post-veraison | 22°C | 71.16 | 11.37 | 48.31 | 94.01 |  |
|  |  | 30°C | 96.71 | 11.37 | 73.86 | 119.56 |  |
|  | Mid-ripening | 22°C | 69.36 | 11.37 | 46.51 | 92.21 |  |
|  |  | 30°C | 64.66 | 11.37 | 41.81 | 87.51 |  |
|  | Ripening | 22°C | 52.11 | 11.37 | 29.26 | 74.96 |  |
|  |  | 30°C | 72.95 | 11.37 | 50.10 | 95.80 |  |
| Threonine | Pre-veraison | 22°C | 384.59 | 140.86 | 101.38 | 667.80 | 0.009 |
|  |  | 30°C | 598.79 | 140.86 | 315.58 | 881.99 |  |
|  | Veraison | 22°C | 606.87 | 140.86 | 323.66 | 890.08 |  |
|  |  | 30°C | 747.29 | 172.51 | 400.43 | 1094.14 |  |
|  | Post-veraison | 22°C | 1264.07 | 140.86 | 980.86 | 1547.27 |  |
|  |  | 30°C | 1440.36 | 140.86 | 1157.15 | 1723.57 |  |
|  | Mid-ripening | 22°C | 858.97 | 140.86 | 575.76 | 1142.18 |  |
|  |  | 30°C | 1390.81 | 140.86 | 1107.60 | 1674.02 |  |
|  | Ripening | 22°C | 1207.14 | 140.86 | 923.93 | 1490.34 |  |
|  |  | 30°C | 1381.60 | 140.86 | 1098.39 | 1664.81 |  |

… AA Table continues

|  |  |  | Mean | Std. Error | 95% Confidence Interval | |  |
| --- | --- | --- | --- | --- | --- | --- | --- |
| Amino acid | Time point | Temperature | (µM) |  | Lower Bound | Upper Bound | *p* value |
| Arginine | Pre-veraison | 22°C | 1004.37 | 366.90 | 266.66 | 1742.07 | 0.225 |
|  |  | 30°C | 1088.20 | 366.90 | 350.50 | 1825.91 |  |
|  | Veraison | 22°C | 1126.58 | 366.90 | 388.88 | 1864.29 |  |
|  |  | 30°C | 1469.93 | 449.36 | 566.43 | 2373.43 |  |
|  | Post-veraison | 22°C | 2281.05 | 366.90 | 1543.35 | 3018.76 |  |
|  |  | 30°C | 2046.62 | 366.90 | 1308.91 | 2784.32 |  |
|  | Mid-ripening | 22°C | 1877.34 | 366.90 | 1139.63 | 2615.04 |  |
|  |  | 30°C | 2793.33 | 366.90 | 2055.63 | 3531.04 |  |
|  | Ripening | 22°C | 2633.57 | 366.90 | 1895.86 | 3371.27 |  |
|  |  | 30°C | 2985.94 | 366.90 | 2248.24 | 3723.65 |  |
| Alanine | Pre-veraison | 22°C | 475.30 | 222.77 | 27.40 | 923.19 | 0.002 |
|  |  | 30°C | 804.97 | 222.77 | 357.07 | 1252.87 |  |
|  | Veraison | 22°C | 724.02 | 222.77 | 276.12 | 1171.91 |  |
|  |  | 30°C | 780.23 | 272.83 | 231.67 | 1328.79 |  |
|  | Post-veraison | 22°C | 1048.22 | 222.77 | 600.32 | 1496.11 |  |
|  |  | 30°C | 1365.70 | 222.77 | 917.80 | 1813.60 |  |
|  | Mid-ripening | 22°C | 713.89 | 222.77 | 265.99 | 1161.79 |  |
|  |  | 30°C | 1337.72 | 222.77 | 889.82 | 1785.61 |  |
|  | Ripening | 22°C | 989.50 | 222.77 | 541.61 | 1437.40 |  |
|  |  | 30°C | 2032.39 | 222.77 | 1584.50 | 2480.29 |  |
| Tyrosine | Pre-veraison | 22°C | 73.07 | 15.02 | 42.87 | 103.28 | 0.22 |
|  |  | 30°C | 86.57 | 15.02 | 56.36 | 116.77 |  |
|  | Veraison | 22°C | 61.80 | 15.02 | 31.60 | 92.01 |  |
|  |  | 30°C | 84.59 | 18.40 | 47.60 | 121.58 |  |
|  | Post-veraison | 22°C | 53.90 | 15.02 | 23.70 | 84.10 |  |
|  |  | 30°C | 74.04 | 15.02 | 43.83 | 104.24 |  |
|  | Mid-ripening | 22°C | 70.58 | 15.02 | 40.38 | 100.78 |  |
|  |  | 30°C | 56.55 | 15.02 | 26.35 | 86.76 |  |
|  | Ripening | 22°C | 53.65 | 15.02 | 23.45 | 83.85 |  |
|  |  | 30°C | 71.78 | 15.02 | 41.58 | 101.98 |  |

… AA Table continues

|  |  |  | Mean | Std. Error | 95% Confidence Interval | |  |
| --- | --- | --- | --- | --- | --- | --- | --- |
| Amino acid | Time point | Temperature | (µM) |  | Lower Bound | Upper Bound | *p* value |
| Valine | Pre-veraison | 22°C | 76.27 | 68.20 | -60.86 | 213.40 | 0.006 |
|  |  | 30°C | 354.17 | 68.20 | 217.04 | 491.30 |  |
|  | Veraison | 22°C | 115.40 | 68.20 | -21.73 | 252.53 |  |
|  |  | 30°C | 148.81 | 83.53 | -19.14 | 316.76 |  |
|  | Post-veraison | 22°C | 154.01 | 68.20 | 16.88 | 291.14 |  |
|  |  | 30°C | 264.96 | 68.20 | 127.83 | 402.09 |  |
|  | Mid-ripening | 22°C | 132.65 | 68.20 | -4.48 | 269.78 |  |
|  |  | 30°C | 221.27 | 68.20 | 84.15 | 358.40 |  |
|  | Ripening | 22°C | 155.94 | 68.20 | 18.81 | 293.07 |  |
|  |  | 30°C | 277.16 | 68.20 | 140.03 | 414.29 |  |
| Methionine | Pre-veraison | 22°C | 21.64 | 9.24 | 3.05 | 40.22 | 0.027 |
|  |  | 30°C | 42.61 | 9.24 | 24.03 | 61.20 |  |
|  | Veraison | 22°C | 23.59 | 9.24 | 5.00 | 42.17 |  |
|  |  | 30°C | 32.83 | 11.32 | 10.07 | 55.59 |  |
|  | Post-veraison | 22°C | 34.53 | 9.24 | 15.94 | 53.11 |  |
|  |  | 30°C | 50.72 | 9.24 | 32.14 | 69.31 |  |
|  | Mid-ripening | 22°C | 36.81 | 9.24 | 18.23 | 55.40 |  |
|  |  | 30°C | 43.25 | 9.24 | 24.67 | 61.84 |  |
|  | Ripening | 22°C | 45.02 | 9.24 | 26.44 | 63.61 |  |
|  |  | 30°C | 60.37 | 9.24 | 41.78 | 78.95 |  |
| Tryptophane | Pre-veraison | 22°C | 142.83 | 28.42 | 85.70 | 199.97 | 0.046 |
|  |  | 30°C | 265.19 | 28.42 | 208.06 | 322.33 |  |
|  | Veraison | 22°C | 180.02 | 28.42 | 122.88 | 237.15 |  |
|  |  | 30°C | 181.02 | 34.80 | 111.05 | 251.00 |  |
|  | Post-veraison | 22°C | 187.49 | 28.42 | 130.35 | 244.62 |  |
|  |  | 30°C | 208.15 | 28.42 | 151.01 | 265.28 |  |
|  | Mid-ripening | 22°C | 110.22 | 28.42 | 53.08 | 167.35 |  |
|  |  | 30°C | 154.15 | 28.42 | 97.02 | 211.29 |  |
|  | Ripening | 22°C | 127.61 | 28.42 | 70.47 | 184.74 |  |
|  |  | 30°C | 128.08 | 28.42 | 70.94 | 185.21 |  |

… AA Table continues

|  |  |  | Mean | Std. Error | 95% Confidence Interval | |  |
| --- | --- | --- | --- | --- | --- | --- | --- |
| Amino acid | Time point | Temperature | (µM) |  | Lower Bound | Upper Bound | p value |
| Phenylalanine | Pre-veraison | 22°C | 81.27 | 24.65 | 31.71 | 130.83 | 0.437 |
|  |  | 30°C | 149.50 | 24.65 | 99.94 | 199.06 |  |
|  | Veraison | 22°C | 70.32 | 24.65 | 20.76 | 119.88 |  |
|  |  | 30°C | 77.47 | 30.19 | 16.77 | 138.17 |  |
|  | Post-veraison | 22°C | 82.12 | 24.65 | 32.56 | 131.68 |  |
|  |  | 30°C | 93.56 | 24.65 | 44.00 | 143.12 |  |
|  | Mid-ripening | 22°C | 81.21 | 24.65 | 31.65 | 130.77 |  |
|  |  | 30°C | 63.65 | 24.65 | 14.09 | 113.21 |  |
|  | Ripening | 22°C | 65.10 | 24.65 | 15.54 | 114.66 |  |
|  |  | 30°C | 58.39 | 24.65 | 8.83 | 107.95 |  |
| Ilenine | Pre-veraison | 22°C | 110.18 | 53.58 | 2.44 | 217.92 | 0.156 |
|  |  | 30°C | 264.28 | 53.58 | 156.54 | 372.02 |  |
|  | Veraison | 22°C | 121.33 | 53.58 | 13.59 | 229.07 |  |
|  |  | 30°C | 140.48 | 65.63 | 8.53 | 272.43 |  |
|  | Post-veraison | 22°C | 145.08 | 53.58 | 37.34 | 252.81 |  |
|  |  | 30°C | 207.98 | 53.58 | 100.24 | 315.71 |  |
|  | Mid-ripening | 22°C | 137.65 | 53.58 | 29.91 | 245.38 |  |
|  |  | 30°C | 145.28 | 53.58 | 37.55 | 253.02 |  |
|  | Ripening | 22°C | 140.60 | 53.58 | 32.86 | 248.34 |  |
|  |  | 30°C | 147.11 | 53.58 | 39.37 | 254.85 |  |
| Lysine | Pre-veraison | 22°C | 23.16 | 5.14 | 12.83 | 33.49 | 0.007 |
|  |  | 30°C | 20.21 | 5.14 | 9.88 | 30.54 |  |
|  | Veraison | 22°C | 29.04 | 5.14 | 18.71 | 39.36 |  |
|  |  | 30°C | 39.18 | 6.29 | 26.53 | 51.83 |  |
|  | Post-veraison | 22°C | 35.16 | 5.14 | 24.83 | 45.49 |  |
|  |  | 30°C | 45.28 | 5.14 | 34.95 | 55.61 |  |
|  | Mid-ripening | 22°C | 32.74 | 5.14 | 22.41 | 43.07 |  |
|  |  | 30°C | 49.18 | 5.14 | 38.85 | 59.51 |  |
|  | Ripening | 22°C | 39.91 | 5.14 | 29.59 | 50.24 |  |
|  |  | 30°C | 53.36 | 5.14 | 43.04 | 63.69 |  |

… AA Table continues

|  |  |  | Mean | Std. Error | 95% Confidence Interval | |  |
| --- | --- | --- | --- | --- | --- | --- | --- |
| Amino acid | Time point | Temperature | (µM) |  | Lower Bound | Upper Bound | *p* value |
| Leucine | Pre-veraison | 22°C | 126.16 | 65.53 | -5.60 | 257.92 | 0.13 |
|  |  | 30°C | 316.65 | 65.53 | 184.89 | 448.40 |  |
|  | Veraison | 22°C | 161.64 | 65.53 | 29.89 | 293.40 |  |
|  |  | 30°C | 224.09 | 80.26 | 62.73 | 385.46 |  |
|  | Post-veraison | 22°C | 174.05 | 65.53 | 42.30 | 305.81 |  |
|  |  | 30°C | 237.62 | 65.53 | 105.86 | 369.37 |  |
|  | Mid-ripening | 22°C | 202.69 | 65.53 | 70.93 | 334.45 |  |
|  |  | 30°C | 198.24 | 65.53 | 66.48 | 329.99 |  |
|  | Ripening | 22°C | 207.36 | 65.53 | 75.61 | 339.12 |  |
|  |  | 30°C | 222.72 | 65.53 | 90.96 | 354.47 |  |
| Proline | Pre-veraison | 22°C | 136.86 | 270.25 | -406.52 | 680.24 | 0.003 |
|  |  | 30°C | 390.59 | 270.25 | -152.79 | 933.97 |  |
|  | Veraison | 22°C | 217.80 | 270.25 | -325.58 | 761.18 |  |
|  |  | 30°C | 290.42 | 330.99 | -375.08 | 955.92 |  |
|  | Post-veraison | 22°C | 946.78 | 270.25 | 403.41 | 1490.16 |  |
|  |  | 30°C | 1144.06 | 270.25 | 600.68 | 1687.44 |  |
|  | Mid-ripening | 22°C | 664.61 | 270.25 | 121.23 | 1207.99 |  |
|  |  | 30°C | 1317.47 | 270.25 | 774.10 | 1860.85 |  |
|  | Ripening | 22°C | 961.47 | 270.25 | 418.09 | 1504.85 |  |
|  |  | 30°C | 2516.12 | 270.25 | 1972.74 | 3059.49 |  |
| Total amino acid | Pre-veraison | 22°C | 6131.92 | 1733.02 | 2647.45 | 9616.38 | 0.015 |
|  |  | 30°C | 11654.91 | 1733.02 | 8170.45 | 15139.38 |  |
|  | Veraison | 22°C | 7547.20 | 1733.02 | 4062.74 | 11031.66 |  |
|  |  | 30°C | 7589.79 | 2122.50 | 3322.21 | 11857.36 |  |
|  | Post-veraison | 22°C | 9614.97 | 1733.02 | 6130.51 | 13099.44 |  |
|  |  | 30°C | 10942.36 | 1733.02 | 7457.89 | 14426.82 |  |
|  | Mid-ripening | 22°C | 7781.76 | 1733.02 | 4297.29 | 11266.22 |  |
|  |  | 30°C | 10906.89 | 1733.02 | 7422.42 | 14391.35 |  |
|  | Ripening | 22°C | 9164.22 | 1733.02 | 5679.75 | 12648.68 |  |
|  |  | 30°C | 13257.51 | 1733.02 | 9773.04 | 16741.97 |  |

Supplementary Table 4. Effect of temperature (22/15°C and 30/15°C) on the concentration of individual phenolics in Pinot noir grapes accumulation from post veraison to ripening in the second experiment (2019/20). CI mean confidence interval at 95%.

|  |  |  | Mean | Std. Error | 95% Confidence Interval |  |  |
| --- | --- | --- | --- | --- | --- | --- | --- |
| Phenolic compound | Time point | Temperature | (mg/g) |  | Lower Bound | Upper Bound | p value |
| Gallic acid | Post-veraison | 22°C | 1 | 0.02 | 0.05 | 0.14 | 0.252 |
|  |  | 30°C | 1.2 | 0.02 | 0.08 | 0.16 |  |
|  | Mid-ripening | 22°C | 1.2 | 0.02 | 0.08 | 0.16 |  |
|  |  | 30°C | 0.8 | 0.02 | 0.04 | 0.11 |  |
|  | Ripening | 22°C | 0.9 | 0.02 | 0.05 | 0.12 |  |
|  |  | 30°C | 0.8 | 0.02 | 0.04 | 0.11 |  |
|  |  | 30°C | 2.8 | 0.05 | 0.17 | 0.39 |  |
| Quercetin | Post-veraison | 22°C | 0.5 | 0.04 | -0.03 | 0.13 | 0.828 |
|  |  | 30°C | 0.5 | 0.03 | -0.02 | 0.11 |  |
|  | Mid-ripening | 22°C | 0.7 | 0.03 | 0 | 0.14 |  |
|  |  | 30°C | 0.7 | 0.03 | 0.02 | 0.13 |  |
|  | Ripening | 22°C | 2.8 | 0.03 | 0.22 | 0.33 |  |
|  |  | 30°C | 2.5 | 0.03 | 0.19 | 0.31 |  |
| Reveratrol | Post-veraison | 22°C | 0.4 | 0.57 | -1.13 | 1.21 | 0.3860 |
|  |  | 30°C | 0.6 | 0.44 | -0.84 | 0.97 |  |
|  | Mid-ripening | 22°C | 0.8 | 0.49 | -0.94 | 1.09 |  |
|  |  | 30°C | 10.8 | 0.4 | 0.26 | 1.91 |  |
|  | Ripening | 22°C | 6.4 | 0.4 | -0.19 | 1.47 |  |
|  |  | 30°C | 5 | 0.4 | -0.32 | 1.33 |  |
| Delphinidin | Post-veraison | 22°C | 3.6 | 0.11 | 0.13 | 0.58 | 0.365 |
|  |  | 30°C | 1.7 | 0.08 | 0 | 0.35 |  |
|  | Mid-ripening | 22°C | 3.9 | 0.09 | 0.2 | 0.59 |  |
|  |  | 30°C | 2.3 | 0.08 | 0.07 | 0.39 |  |
|  | Ripening | 22°C | 0.5 | 0.08 | -0.11 | 0.21 |  |
|  |  | 30°C | 0.8 | 0.08 | -0.07 | 0.24 |  |
| Malvidin | Post-veraison | 22°C | 25.8 | 0.46 | 1.63 | 3.53 | 0.273 |
|  |  | 30°C | 19.8 | 0.36 | 1.25 | 2.72 |  |
|  | Mid-ripening | 22°C | 30.3 | 0.4 | 2.21 | 3.86 |  |
|  |  | 30°C | 17.3 | 0.33 | 1.06 | 2.41 |  |
|  | Ripening | 22°C | 15.8 | 0.33 | 0.91 | 2.26 |  |
|  |  | 30°C | 14.3 | 0.33 | 0.75 | 2.1 |  |
| Petunidin | Post-veraison | 22°C | 14.2 | 0.4 | 0.59 | 2.25 | 0.481 |
|  |  | 30°C | 7.9 | 0.31 | 0.15 | 1.44 |  |
|  | Mid-ripening | 22°C | 15.8 | 0.35 | 0.86 | 2.3 |  |
|  |  | 30°C | 8.2 | 0.28 | 0.23 | 1.41 |  |
|  | Ripening | 22°C | 4.4 | 0.28 | -0.15 | 1.02 |  |
|  |  | 30°C | 3.7 | 0.28 | -0.22 | 0.95 |  |
| Peonidin | Post-veraison | 22°C | 8.2 | 1.36 | -1.99 | 3.63 | 0.407 |
|  |  | 30°C | 6.5 | 1.05 | -1.52 | 2.83 |  |
|  | Mid-ripening | 22°C | 14.8 | 1.18 | -0.95 | 3.91 |  |
|  |  | 30°C | 37.9 | 0.96 | 1.8 | 5.78 |  |
|  | Ripening | 22°C | 57.7 | 0.96 | 3.78 | 7.75 |  |
|  |  | 30°C | 55 | 0.96 | 3.52 | 7.49 |  |
| Total anthocyanin | Post-veraison | 22°C | 518.3 | 13.33 | 24.31 | 79.35 | 0.7590 |
|  |  | 30°C | 360.2 | 10.33 | 14.7 | 57.34 |  |
|  | Mid-ripening | 22°C | 648.8 | 11.55 | 41.05 | 88.72 |  |
|  |  | 30°C | 657 | 9.43 | 46.23 | 85.16 |  |
|  | Ripening | 22°C | 784.2 | 9.43 | 58.96 | 97.88 |  |
|  |  | 30°C | 738.3 | 9.43 | 54.37 | 93.29 |  |

Supplementary Table 5. Effect of temperature (22/15°C and 30/15°C) on the content of individual phenolics in Pinot noir grapes from pre-veraison to ripening in the second experiment (2019/20). CI mean confidence interval at 95%.

|  |  |  | Mean | Std. Error | 95% Confidence Interval | |  |
| --- | --- | --- | --- | --- | --- | --- | --- |
| Phenolic compound | Time point | Temperature | (mg/g) |  | Lower Bound | Upper Bound | *p* value |
| Epicatechin | Pre-veraison | 22°C | 83.89 | 1.05 | 6.28 | 10.50 | 0.004 |
|  |  | 30°C | 107.23 | 1.05 | 8.62 | 12.83 |  |
|  | Veraison | 22°C | 102.33 | 1.05 | 8.13 | 12.34 |  |
|  |  | 30°C | 64.04 | 1.28 | 3.82 | 8.98 |  |
|  | Post-veraison | 22°C | 57.01 | 1.15 | 3.39 | 8.01 |  |
|  |  | 30°C | 96.70 | 1.05 | 7.56 | 11.78 |  |
|  | Mid-ripening | 22°C | 63.03 | 1.28 | 3.72 | 8.88 |  |
|  |  | 30°C | 31.03 | 1.05 | 1.00 | 5.21 |  |
|  | Ripening | 22°C | 16.99 | 1.05 | -0.41 | 3.81 |  |
|  |  | 30°C | 14.30 | 1.05 | -0.68 | 3.54 |  |
| Epicatechin gallate | Pre-veraison | 22°C | 33.76 | 0.37 | 2.64 | 4.11 | 0.617 |
|  |  | 30°C | 36.46 | 0.37 | 2.91 | 4.38 |  |
|  | Veraison | 22°C | 29.51 | 0.37 | 2.21 | 3.69 |  |
|  |  | 30°C | 21.78 | 0.45 | 1.28 | 3.08 |  |
|  | Post-veraison | 22°C | 8.57 | 0.40 | 0.05 | 1.66 |  |
|  |  | 30°C | 12.33 | 0.37 | 0.50 | 1.97 |  |
|  | Mid-ripening | 22°C | 5.57 | 0.45 | -0.35 | 1.46 |  |
|  |  | 30°C | 3.17 | 0.37 | -0.42 | 1.05 |  |
|  | Ripening | 22°C | 0.38 | 0.37 | -0.70 | 0.77 |  |
|  |  | 30°C | 0.33 | 0.37 | -0.70 | 0.77 |  |
| Procyanidin B2 | Pre-veraison | 22°C | 3.93 | 0.05 | 0.30 | 0.49 | 0.127 |
|  |  | 30°C | 4.43 | 0.05 | 0.35 | 0.54 |  |
|  | Veraison | 22°C | 4.08 | 0.05 | 0.31 | 0.50 |  |
|  |  | 30°C | 3.06 | 0.06 | 0.19 | 0.42 |  |
|  | Post-veraison | 22°C | 2.85 | 0.05 | 0.18 | 0.39 |  |
|  |  | 30°C | 4.26 | 0.05 | 0.33 | 0.52 |  |
|  | Mid-ripening | 22°C | 3.17 | 0.06 | 0.20 | 0.43 |  |
|  |  | 30°C | 2.48 | 0.05 | 0.15 | 0.34 |  |
|  | Ripening | 22°C | 2.86 | 0.05 | 0.19 | 0.38 |  |
|  |  | 30°C | 2.83 | 0.05 | 0.19 | 0.38 |  |

… Phenolics table continues

|  |  |  | Mean | Std. Error | 95% Confidence Interval | |  |
| --- | --- | --- | --- | --- | --- | --- | --- |
| Phenolic compound | Time point | Temperature | (mg/g) |  | Lower Bound | Upper Bound | *p* value |
| Caftaric acid | Pre-veraison | 22°C | 5.98 | 0.05 | 0.49 | 0.70 | 0.044 |
|  |  | 30°C | 8.20 | 0.05 | 0.72 | 0.92 |  |
|  | Veraison | 22°C | 6.02 | 0.05 | 0.50 | 0.71 |  |
|  |  | 30°C | 5.72 | 0.06 | 0.44 | 0.70 |  |
|  | Post-veraison | 22°C | 3.68 | 0.06 | 0.25 | 0.48 |  |
|  |  | 30°C | 3.20 | 0.05 | 0.22 | 0.42 |  |
|  | Mid-ripening | 22°C | 3.13 | 0.06 | 0.19 | 0.44 |  |
|  |  | 30°C | 2.26 | 0.05 | 0.12 | 0.33 |  |
|  | Ripening | 22°C | 3.80 | 0.05 | 0.28 | 0.48 |  |
|  |  | 30°C | 3.76 | 0.05 | 0.27 | 0.48 |  |
| Catechin | Pre-veraison | 22°C | 166.87 | 3.30 | 10.04 | 23.34 | 0.446 |
|  |  | 30°C | 162.14 | 3.30 | 9.56 | 22.87 |  |
|  | Veraison | 22°C | 195.72 | 3.30 | 12.92 | 26.22 |  |
|  |  | 30°C | 141.74 | 4.04 | 6.03 | 22.32 |  |
|  | Post-veraison | 22°C | 99.51 | 3.62 | 2.66 | 17.24 |  |
|  |  | 30°C | 167.18 | 3.30 | 10.07 | 23.37 |  |
|  | Mid-ripening | 22°C | 121.43 | 4.04 | 4.00 | 20.29 |  |
|  |  | 30°C | 133.33 | 3.30 | 6.68 | 19.98 |  |
|  | Ripening | 22°C | 127.48 | 3.30 | 6.10 | 19.40 |  |
|  |  | 30°C | 86.24 | 3.30 | 1.97 | 15.28 |  |
| Procyanidin B1 | Pre-veraison | 22°C | 5.67 | 0.08 | 0.41 | 0.72 | 0.017 |
|  |  | 30°C | 6.70 | 0.08 | 0.52 | 0.82 |  |
|  | Veraison | 22°C | 6.17 | 0.08 | 0.46 | 0.77 |  |
|  |  | 30°C | 4.30 | 0.09 | 0.24 | 0.62 |  |
|  | Post-veraison | 22°C | 5.02 | 0.08 | 0.33 | 0.67 |  |
|  |  | 30°C | 8.63 | 0.08 | 0.71 | 1.02 |  |
|  | Mid-ripening | 22°C | 7.37 | 0.09 | 0.55 | 0.93 |  |
|  |  | 30°C | 6.22 | 0.08 | 0.47 | 0.78 |  |
|  | Ripening | 22°C | 6.53 | 0.08 | 0.50 | 0.81 |  |
|  |  | 30°C | 7.01 | 0.08 | 0.55 | 0.86 |  |
| Caffeic acid | Pre-veraison | 22°C | 0.67 | 0.01 | 0.05 | 0.08 | 0.002 |
|  |  | 30°C | 1.08 | 0.01 | 0.09 | 0.12 |  |
|  | Veraison | 22°C | 0.73 | 0.01 | 0.06 | 0.09 |  |
|  |  | 30°C | 0.64 | 0.01 | 0.05 | 0.08 |  |
|  | Post-veraison | 22°C | 0.48 | 0.01 | 0.03 | 0.06 |  |
|  |  | 30°C | 0.46 | 0.01 | 0.03 | 0.06 |  |
|  | Mid-ripening | 22°C | 0.41 | 0.01 | 0.02 | 0.06 |  |
|  |  | 30°C | 0.24 | 0.01 | 0.01 | 0.04 |  |
|  | Ripening | 22°C | 0.46 | 0.01 | 0.03 | 0.06 |  |
|  |  | 30°C | 0.48 | 0.01 | 0.03 | 0.06 |  |
| Epigallocatechin | Pre-veraison | 22°C | 1.66 | 0.27 | -0.38 | 0.71 | 0.159 |
|  |  | 30°C | 2.07 | 0.27 | -0.34 | 0.75 |  |
|  | Veraison | 22°C | 1.66 | 0.27 | -0.38 | 0.71 |  |
|  |  | 30°C | 1.34 | 0.33 | -0.53 | 0.80 |  |
|  | Post-veraison | 22°C | 1.33 | 0.30 | -0.46 | 0.73 |  |
|  |  | 30°C | 2.48 | 0.27 | -0.30 | 0.79 |  |
|  | Mid-ripening | 22°C | 2.29 | 0.33 | -0.44 | 0.90 |  |
|  |  | 30°C | 12.88 | 0.27 | 0.74 | 1.83 |  |
|  | Ripening | 22°C | 30.91 | 0.27 | 2.55 | 3.64 |  |
|  |  | 30°C | 26.76 | 0.27 | 2.13 | 3.22 |  |
| Total phenolics | Pre-veraison | 22°C | 3025.62 | 43.27 | 215.41 | 389.72 | 0.185 |
|  |  | 30°C | 3287.13 | 43.27 | 241.56 | 415.87 |  |
|  | Veraison | 22°C | 3462.72 | 43.27 | 259.11 | 433.43 |  |
|  |  | 30°C | 2426.97 | 53.00 | 135.95 | 349.44 |  |
|  | Post-veraison | 22°C | 1796.68 | 47.40 | 84.19 | 275.14 |  |
|  |  | 30°C | 2974.30 | 43.27 | 210.27 | 384.59 |  |
|  | Mid-ripening | 22°C | 2090.22 | 53.00 | 102.28 | 315.77 |  |
|  |  | 30°C | 2039.35 | 43.27 | 116.78 | 291.09 |  |
|  | Ripening | 22°C | 1994.33 | 43.27 | 112.28 | 286.59 |  |
|  |  | 30°C | 1500.20 | 43.27 | 62.86 | 237.18 |  |

**Supplementary Table 6*.*** Effect of temperature (22/15°C and 30/15°C) on the concentration of significant individual phenolics in Pinot noir grapes at each harvesting time point from pre-veraison to ripening in the second experiment (2019/20). CI mean confidence interval at 95%.

| Time point | Pre-veraison | | Veraison | | | Post-veraison | | Mid-ripening | | Harvest | |
| --- | --- | --- | --- | --- | --- | --- | --- | --- | --- | --- | --- |
| **Epicatechin** | | | | | | | | | | | |
| Temperature | 22°C/15°C | 30°C/15°C | 22°C/15°C | | 30°C/15°C | 22°C/15°C | 30°C/15°C | 22°C/15°C | 30°C/15°C | 22°C/15°C | 30°C/15°C |
| Mean (mg/g) | 83.89 | 107.23 | 102.33 | | 64.04 | 57.01 **b** | 96.70 **a** | 63.03 | 31.03 | 16.99 | 14.30 |
| CI-Lower Bound | 6.28 | 8.62 | 8.13 | | 3.82 | 3.39 | 7.56 | 3.72 | 1.00 | -0.41 | -0.68 |
| CI-Upper Bound | 10.50 | 12.83 | 12.34 | | 8.98 | 8.01 | 11.78 | 8.88 | 5.21 | 3.81 | 3.54 |
| *p* value | 0.284 | | 0.063 | | | 0.008 | | 0.133 | | 0.725 | |
| **Procyanidin** | | | | | | | | | | | |
| Mean (mg/g) | 5.67 | 6.70 | 6.17 **a** | | 4.30 **b** | 5.02 **b** | 8.63 **a** | 7.37 | 6.22 | 6.53 | 7.01 |
| CI-Lower Bound | 0.41 | 0.52 | 0.46 | | 0.24 | 0.33 | 0.71 | 0.55 | 0.47 | 0.50 | 0.55 |
| CI-Upper Bound | 0.72 | 0.82 | 0.77 | | 0.62 | 0.67 | 1.02 | 0.93 | 0.78 | 0.81 | 0.86 |
| *p* value | 0.337 | | | 0.045 | | 0.025 | | 0.676 | | 0.713 | |
| **Caftaric acid** | | | | | | | | | | | |
| Mean (mg/g) | 5.98 **b** | 8.20 **a** | 6.02 | | 5.72 | 3.68 | 3.20 | 3.13 | 2.26 | 3.80 | 3.76 |
| CI-Lower Bound | 0.49 | 0.72 | 0.50 | | 0.44 | 0.25 | 0.22 | 0.19 | 0.12 | 0.28 | 0.27 |
| CI-Upper Bound | 0.70 | 0.92 | 0.71 | | 0.70 | 0.48 | 0.42 | 0.44 | 0.33 | 0.48 | 0.48 |
| *p* value | 0.006 | | | 0.801 | | 0.654 | | 0.064 | | 0.945 | |
| **Caffeic acid** | | | | | | | | | | | |
| Mean (mg/g) | 0.67 **b** | 1.08 **a** | 0.73 | | 0.64 | 0.48 | 0.46 | 0.41 **b** | 0.34 **a** | 0.46 | 0.48 |
| CI-Lower Bound | 0.05 | 0.09 | 0.06 | | 0.05 | 0.03 | 0.03 | 0.02 | 0.01 | 0.03 | 0.03 |
| CI-Upper Bound | 0.08 | 0.12 | 0.09 | | 0.08 | 0.06 | 0.06 | 0.06 | 0.04 | 0.06 | 0.06 |
| *p* value | 0.003 | | | 0.589 | | 0.963 | | 0.061 | | 0.810 | |
